# Beyond Pandemic Preparedness: Reframing Protracted War Against Infectious Diseases

**DOI:** 10.3390/v18030373

**Published:** 2026-03-17

**Authors:** Ming Zheng

**Affiliations:** 1 Human Disease Continuum Project Consortium (HDCPC), Beijing, China; mmzheng@fmmu.edu.cn or zhengming_china@163.com; 2 Beijing Institute of Basic Medical Sciences, 27 Taiping Road, Beijing 100850, China

Recent changes in the strategic language of the US National Institute of Allergy and Infectious Diseases (NIAID), including the removal of “biodefense” and “pandemic preparedness,” signal a substantive reordering of infectious-disease priorities. This editorial argues that preparedness should be understood not as a slogan, but as a durable set of capacities spanning surveillance, pathogen characterization, countermeasure development, clinical readiness, and management of post-infectious sequelae. A narrow focus on the immediate infectious burden, while neglecting the broader scientific and clinical infrastructure, is insufficient to address the long-term consequences of infection. This tension is especially visible in virus-associated autoimmunity. Viral infections can precipitate, unmask, or amplify autoimmune disease long after acute infection has resolved, making short-term metrics inadequate for capturing their true health impact. Evidence linking the Epstein–Barr virus to multiple sclerosis, enteroviruses to type 1 diabetes, and SARS-CoV-2 to post-acute immune-mediated disorders underscores the need for a common, patient-centered framework integrating infectious diseases into a unified disease continuum of viral autoimmunity (Figure 1).

Recently, the US National Institute of Allergy and Infectious Diseases (NIAID) staff were instructed to remove the terms “biodefense” and “pandemic preparedness” from NIAID web pages amid a wider restructuring and a stated intent to deprioritize those research areas. This is not a trivial edit. Language on an institute’s public-facing portfolio is a policy instrument: it signals how grant lines may be rewritten, how review panels may be reconstituted, and how “mission” will be interpreted by applicants, Congress, and partner agencies [1]. At the same time, NIAID has articulated a “new vision” that argues for pivoting away from an older three-part model, HIV, biodefense/pandemic preparedness, and “everything else,” toward two pillars: infectious diseases and immunology, with more urgency around conditions affecting Americans “today,” and an explicit claim that NIAID’s pandemic-era work “neither prevented the pandemic” nor prevented unusually high US excess mortality [2].

NIAID’s shift is occurring under intense political constraints and amid an acknowledged “breach of trust” that shape both budgets and narratives. In that environment, “pandemic preparedness” and “biodefense” are not neutral descriptors: they are politically charged symbols, associated with lockdown politics, lab-origin debates, “gain-of-function” controversy, and expansive emergency powers during the COVID-19 pandemic. NIAID’s new vision also commits to strict adherence to new regulatory frameworks on “dangerous gain-of-function research,” referencing Executive Order 14292 from May 2025 [3]. Together, politicization and tightened oversight create strong incentives to narrow mission language, even when the underlying scientific needs persist. This matters because biodefense and high-consequence pathogen work are frequently (and sometimes incorrectly) conflated with high-risk experimentation. Tightening the risk envelope may be scientifically prudent, but it can also incentivize a rhetorical retreat from “biodefense” as a label—even where safer biodefense activities (surveillance, diagnostics, platform vaccines) remain essential.

Beyond politics, the disappearance of “pandemic preparedness” is analytically important because it denotes a retreat from a previously operationalized strategy, not merely the retirement of a slogan. NIAID previously treated pandemic preparedness as a defined set of goals and infrastructure, which included a Pandemic Preparedness Plan and a strategy that prioritizes prototype pathogens and “high-priority pathogens” [4]. Related investments include networks organized around prototype pathogen families, explicitly justified as groundwork for faster countermeasure development before a crisis. The rebranding debate is therefore less about whether NIAID should study tomorrow’s threats and more about how to preserve minimum readiness while shifting rhetoric and incentives toward measurable health gains. In this light, the defensible corrective is not “stop preparedness”; it is to avoid overconfident claims while sustaining investment in broadly adaptable, generalizable capacities against biological threats.

NIAID’s “new vision” explicitly places long COVID and related post-infectious syndromes within its immunology remit. The under-discussed implication is that other infections also generate long tails. Studies of post-acute sequelae show that influenza survivors can experience sustained complications; COVID-19 often confers higher risk, but the “post-acute” frame applies beyond one virus [5,6]. A burden-based agenda can therefore strengthen preparedness if “burden” is defined as total health impact over time, not only acute hospitalizations and deaths. Reframing preparedness as reducing long-run infectious-disease burden—including chronic sequelae—offers a way to align readiness with tangible, patient-centered outcomes, a linkage that pandemic-era public messaging often struggled to establish.

However, a pivot toward post-infectious prioritization becomes hazardous if it precipitates an availability collapse of capabilities whose value is most apparent only during acute crises. Pandemic preparedness is not a unitary construct; rather, it comprises an interconnected continuum of capacities spanning upstream functions—surveillance, pathogen discovery and characterization, biocontainment operations, prototype libraries, animal models, assay development, pre-positioned clinical-trial networks, rapid contracting mechanisms, and manufacturing partnerships—and downstream functions, including clinical care pathways, rehabilitation, and long-term management of chronic post-infectious sequelae. Many upstream elements are capital- and labor-intensive and depend on highly specialized expertise and safety culture. Consequently, if reprioritization leads to workforce attrition, diminished operational tempo, or degradation of infrastructure, preparedness capacity may erode more rapidly than it can be reconstituted. Downstream improvements (for example, enhanced care for long COVID) and upstream readiness are therefore complementary rather than substitutable; substituting downstream care for upstream preparedness would likely increase the probability and magnitude of catastrophic outcomes in future outbreaks and other biological emergencies.

Viewed from this perspective, the apparent divide between pandemic preparedness and present-tense burden is false in one especially important domain: virus-associated autoimmunity. Viral infections do not necessarily end when viremia clears or PCR turns negative; for a subset of hosts, they initiate months- to years-long immunologic programs that precipitate, unmask, or amplify disease directed at self. Preparedness frameworks that only consider acute admissions, near-term deaths, and immediate economic disruption will therefore underestimate the true burden of infection. The epidemiologic literature now makes that omission increasingly difficult to defend. The Epstein–Barr virus (EBV) infection precedes and markedly elevates the risk of multiple sclerosis; enteroviruses are consistently associated with islet autoimmunity and type 1 diabetes; and post-COVID cohort and meta-analytic studies indicate increased incidence of a range of immune-mediated disorders after SARS-CoV-2 infection. In this sense, neglecting post-viral autoimmunity is not peripheral to infectious-disease control; it results in an underestimation of the total burden attributable to infection. [7,8,9,10].

What has changed most in recent years is not merely the quantity of evidence, but its quality. The field is moving, albeit unevenly, from associative epidemiology toward mechanism. In multiple sclerosis, longitudinal serology in more than 10 million young adults on active duty in the US military showed that EBV seroconversion preceded disease and increased risk 32-fold, while mechanistic work identified high-affinity molecular mimicry between the EBV antigen EBNA1 and GlialCAM, thereby providing structural and functional evidence for a plausible pathogenic bridge from infection to central nervous system autoimmunity. More recently, single-cell viro-immunologic profiling in systemic lupus erythematosus has shown that EBV can reprogram autoreactive B cells into antigen-presenting cells that propagate systemic autoimmune circuits. These studies do not settle causality for every virus–disease pairing, but they materially alter the burden of proof: “virus-associated autoimmunity” can no longer be relegated to a residual category of unexplained correlation [7,11,12].

Type 1 diabetes illustrates a complementary paradigm. Here, the strongest signal centers not on a ubiquitous latent herpesvirus but on enteroviruses, particularly Enterovirus B species, and on the possibility that persistent low-grade pancreatic infection helps sustain beta-cell stress, neoantigen release, and progressive immune injury. A 2023 meta-analysis of molecular detection studies found robust associations between enteroviruses and both islet autoimmunity and type 1 diabetes, while subsequent translational analyses have gone further by framing antiviral therapy and vaccine development not as speculative adjuncts, but as testable disease-modifying strategies. The implication for infectious-disease policy is profound: some conditions classified clinically as “autoimmune” may prove, in biologically meaningful subsets of patients, to be post-viral diseases with long latency, layered host susceptibility, and narrow therapeutic windows [8,13].

This is also why long COVID or long flu should not be treated as an exceptional case whose relevance ends with one pandemic [14]. The broader lesson is that viral outbreaks generate delayed immunologic phenotypes that are clinically heterogeneous, mechanistically mixed, and administratively easy to miss [5,15]. In long COVID, systematic-review evidence indicates recurrent associations between autoantibodies and persistent symptoms, yet also underscores substantial heterogeneity in case definitions, sampling windows, and assay platforms. That combination—signal plus heterogeneity—is precisely what should trigger more disciplined, not less ambitious, investigation. It argues for harmonized longitudinal cohorts, serial biospecimen collection, deep immunophenotyping, and a careful distinction between pathogenic autoimmunity, epiphenomenal autoreactivity, viral persistence, microvascular injury, and tissue-repair programs [10,16].

Accordingly, the research agenda linking infectious diseases to immunology is not narrower than preparedness; it is one of preparedness’s most durable justifications. To identify which infections trigger autoimmune disease, in whom, and by what mechanism, one needs precisely the capacities that are easiest to lose in periods of political retrenchment and hardest to rebuild during crisis: prospective cohorts established before exposure, interoperable registries, validated serologic and cellular assays, biobank samples spanning the acute-to-chronic transition, virology and containment expertise where appropriate, and clinical networks capable of embedding mechanistic studies into outbreak response. These are not luxuries appended to “real” infectious-disease work. They are the connective tissue between acute response and long-term burden reduction. Retiring the vocabulary of preparedness does not retire the infrastructure on which preparedness depends [1,2,3,4].

Accordingly, the interplay between viral infections and autoimmune diseases emerges as a particularly timely subject of inquiry, not despite the current policy climate, but precisely in light of it. This necessitates contributions that move beyond the artificial dichotomy between acute infection control and chronic sequelae, and instead integrate infectious diseases into a unified disease continuum of viral autoimmunity [14]. Particularly important will be studies that integrate epidemiology with mechanism, pair single-cell or spatial methods with clinically annotated cohorts, and translate virologic insight into biomarkers and intervention—whether antiviral, immunomodulatory, tolerogenic, or vaccine-based. If the next phase of infectious-disease research is to be judged by tangible benefit to patients, then few areas better satisfy that standard than the effort to explain how antiviral responses become misdirected against self, and how that misdirection might be predicted, prevented, or reversed.

## Figures and Tables

**Figure 1:**
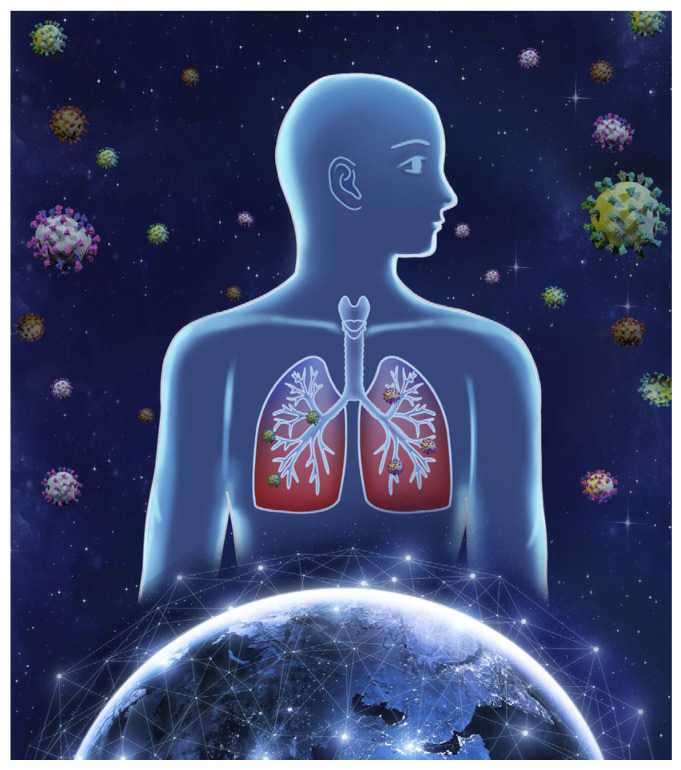
Rethinking pandemic preparedness with the long-tail burden of infectious diseases. Pandemic preparedness should extend beyond a narrow focus on acute outbreaks to recognize infectious diseases as drivers of enduring clinical burden, including chronic post-infectious and immune-mediated disorders. This reframing supports an agenda centered on sustained scientific capacity, clinical infrastructure, and long-term burden reduction.
